# DNA Binding Properties of the Actin-Related Protein Arp8 and Its Role in DNA Repair

**DOI:** 10.1371/journal.pone.0108354

**Published:** 2014-10-09

**Authors:** Akihisa Osakabe, Yuichiro Takahashi, Hirokazu Murakami, Kenji Otawa, Hiroaki Tachiwana, Yukako Oma, Hitoshi Nishijima, Kei-ich Shibahara, Hitoshi Kurumizaka, Masahiko Harata

**Affiliations:** 1 Laboratory of Molecular Biology, Graduate School of Agricultural Science, Tohoku University, Sendai, Japan; 2 Laboratory of Structural Biology, Graduate School of Advanced Science and Engineering, Waseda University, Tokyo, Japan; 3 Department of Integrated Genetics, National Institute of Genetics, Mishima, Japan; George Mason University, United States of America

## Abstract

Actin and actin-related proteins (Arps), which are members of the actin family, are essential components of many of these remodeling complexes. Actin, Arp4, Arp5, and Arp8 are found to be evolutionarily conserved components of the INO80 chromatin remodeling complex, which is involved in transcriptional regulation, DNA replication, and DNA repair. A recent report showed that Arp8 forms a module in the INO80 complex and this module can directly capture a nucleosome. In the present study, we showed that recombinant human Arp8 binds to DNAs, and preferentially binds to single-stranded DNA. Analysis of the binding of adenine nucleotides to Arp8 mutants suggested that the ATP-binding pocket, located in the evolutionarily conserved actin fold, plays a regulatory role in the binding of Arp8 to DNA. To determine the cellular function of Arp8, we derived tetracycline-inducible Arp8 knockout cells from a cultured human cell line. Analysis of results obtained after treating these cells with aphidicolin and camptothecin revealed that Arp8 is involved in DNA repair. Together with the previous observation that Arp8, but not γ-H2AX, is indispensable for recruiting INO80 complex to DSB in human, results of our study suggest an individual role for Arp8 in DNA repair.

## Introduction

Chromatin structure governs genome function, including transcription, DNA damage repair, and replication. The chromatin structure, in its default state, limits the accessibility of DNA binding factors. So, in order for gene expression and DNA repair to take place, chromatin must open up for these factors. Chromatin remodeling complexes are known to play a major role in chromatin opening. Consequently, their activity and recruitment to chromatin must be tightly regulated for exercising proper genome functioning. These remodeling complexes contain multiple regulatory subunits. Thus, to understand the epigenetic regulatory mechanisms of these complexes, it is imperative to know the properties of their regulatory subunits.

Several members of the actin family of proteins, which are evolutionarily conserved, are essential components of these chromatin remodeling complexes [1], [2]. The actin family consists of conventional actin and other evolutionarily and structurally similar actin-related proteins (Arps). Although only a portion of actin is found in the nucleus, some of the Arps are predominantly localized in the nucleus. These nuclear Arps, in most cases together with actin, are known to be essential components of various chromatin modulating complexes. For example, the INO80 chromatin remodeling complex, which is evolutionarily conserved from yeast to man, have been reported to contain actin and three Arps (Arp4, Arp5, and Arp8). Actin and Arps share the evolutionarily conserved actin fold, which contains the ATP-binding pocket at the center. A model has been proposed, wherein any structural change in the actin fold of actin or an Arp, occurred as a result of binding of an adenine nucleotide (ATP/ADP) to this ATP-binding pocket, contributes to the regulation of cellular functions of these proteins, including polymerization of actin, and also probably assembly of actin and Arps into chromatin remodeling complexes [1], [3], [4], [5].

Two major roles have been proposed for the nuclear Arps in chromatin remodeling and histone modification complexes. First, Arps are responsible for recruiting the complexes to chromatin. Indeed, Arp4 and Arp8 have been shown to bind to core histones [6], [7], [8], [9], [10]. It has been shown that the yeast Arp8 binds to a 30 bp long DNA with low affinity (in the micromolar range), whereas the human Arp8 binds to the same 30-bp long DNA with about 3-fold less affinity [9]. Arp5 is also required for the recruitment of INO80 complex to chromatin, although direct binding of Arp5 to chromatin has not been detected so far (Chen et al., 2014; Shen et al., 2003). Second, it has been shown that nuclear Arps regulate the ATPase activity of the Snf2-type ATPase of the chromatin remodeling complexes (Matsuda et al., 2010; Wu et al., 2003; Wu et al., 2005). In yeast, Arp5 and Arp8 seem to regulate the ATPase activity of INO80 by different mechanisms. Thus, the ATPase activity of INO80 lacking the Arp8 was not stimulated by DNA, but was simulated only by the nucleosome core particle, whereas the ATPase activity of INO80 lacking the Arp5 was stimulated by DNA, but was not stimulated by the nucleosome [11].

The INO80 complex binds to selected regions of the genome, including the 5′ and 3′ regions of the open reading frames of genes, and regulates gene expression [12], [13]. In addition, the INO80 complex is recruited to double-strand breaks (DSBs) [14], [15] and to stalled replication forks [16], and is involved in maintaining the genome integrity by promoting the repair processes and restarting the replication at the stalled fork. Both in budding yeast and human, the INO80 complex is required during the DSB repair for effective DNA end resection [14], [15]. Since DNA end resection is an early event that take place during the homologous recombination (HR) repair process, it is believed that the INO80 complex assists the function of an endonuclease through the remodeling of nucleosomes proximal to DSB.

In humans, defects in the maintenance of genome stability can lead to cancer development and progression. Interestingly, by using a RNA interference assay, it was observed that among all the tested subunits only Arp8 was indispensable for recruiting the INO80 complex to DSB in human cells [17]. Thus, to understand the underlying molecular basis of multiple function of the INO80 complex in gene expression and genome integrity, it would be necessary to analyze the biochemical properties of human Arp8 and determine the phenotype of human cells lacking Arp8. In the present study, we purified and characterized the bacterially expressed human Arp8 and also established a tetracycline (tet)-inducible Arp8-knockout human cell line. We found that the purified human Arp8 possessed ssDNA-binding activity, and therefore, we subsequently examined its possible role in HR repair of human genome.

## Results

### Preparation of recombinant human Arp8 and its deletants

**Figure 1:**
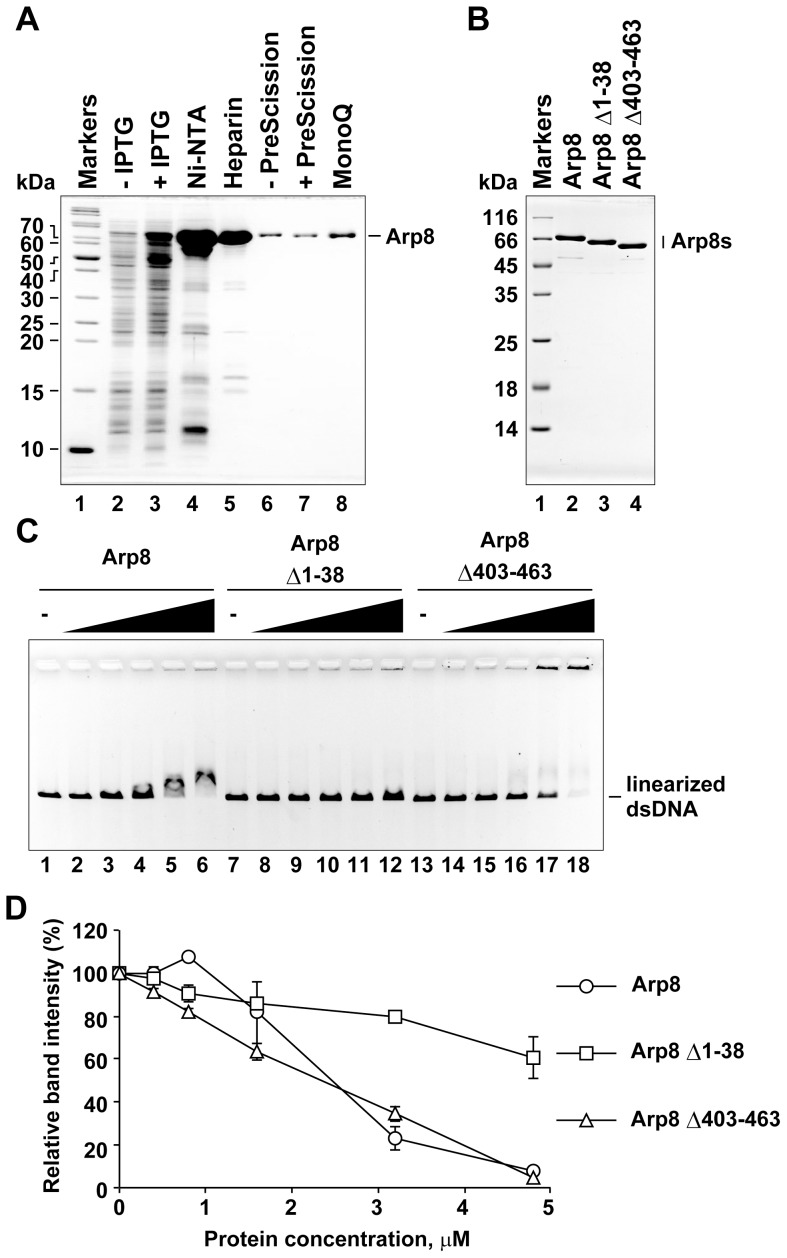
Double-stranded DNA binding activity of purified human Arp8 and its mutants. (A) Purification of human Arp8. Protein fractions from each purification step were analyzed by SDS-PAGE (gel was stained with Coomassie Brilliant Blue). Lane 1, molecular weight markers. Lanes 2 and 3, whole cell lysates before and after induction with IPTG, respectively. Lanes 4 and 5, peak fractions from Ni-NTA agarose and Heparin Sepharose columns, respectively. Lanes 6 and 7, Heparin Sepharose fraction before and after treatment with PreScission protease (removal of His6 tag). Lane 8, peak fraction from MonoQ column. (B) Purified wild-type and deletion mutants (deletants) of Arp8. Lane 1, molecular weight markers. Lanes 2–4, purified Arp8, Arp8 Δ1–38 (N-terminal deletion), and Arp8 Δ403–463 (insertion IV deletion), respectively. (C) dsDNA binding activities of Arp8 and its deletants. Bindings of Arp8 (lanes 2–6), Arp8 Δ1–38 (lanes 8–12), and Arp8 Δ403–463 (lanes 14–18) to linearized φX174 were examined at various protein concentrations: 0 µM (lanes 1, 7, and 13), 0.4 µM (lanes 2, 8, and 14), 0.8 µM (lanes 3, 9, and 15), 1.6 µM (lanes 4, 10, and 16), 3.2 µM (lanes 5, 11, and 17), and 4.8 µM (lanes 6, 12, and 18). (D)Intensity of the unbound DNA in each lane of panel C was quantified and then plotted as relative intensity (%) with respect to that of the unbound DNA from the control (no protein added control) lane.

To analyze the biochemical properties of human Arp8, we purified recombinant human Arp8 after expressing it in *E. coli* cells as a His_6_-tagged protein (Fig. 1A). During the purification process, the His_6_ tag was removed by treating with PreScission protease to obtain purified recombinant Arp8 without the His_6_ tag. A characteristic feature of Arps is the presence of specific insertions in the actin fold, a core structure common to both actin and Arps. The human Arp8 contains an extension in the N-terminal end. In addition, it has multiple insertions, of which insert IV is the largest (Fig. S1) [7], [9], [10], [18]. To analyze the functions of the N-terminal extension and insertion IV, we created and purified an Arp8 deletion mutant lacking the N-terminal amino acids 1 to 38 (Arp8 Δ1-38; Fig. 1B and Fig. S1) and another Arp8 deletion mutant lacking the insertion IV (deletion of amino acids 403 to 463; Arp8 Δ403-463; Fig. 1B and Fig. S1).

### Arp8 binds to double-stranded DNA

The N-terminal extension of the human Arp8 is abundant in basic amino acids. We previously analyzed this N-terminal sequence using a software and predicted that it might bind to DNA [19]. It was reported earlier that Arp8 associated with a synthetic 30-bp double-stranded DNA (dsDNA), albeit with very low affinity, and the N-terminal extension was not necessary for the association [9]. We expected that the human Arp8 would form stable complexes with DNAs and the N-terminal extension is involved in this DNA binding process. Gel shift analysis revealed that the full length Arp8 bound to a linearized plasmid DNA (Fig. 1C, lanes 1 to 6). Arp8 also bound to nicked-circular and closed-circular DNAs (Fig. S2). The DNA binding activity of Arp8 is consistent with the earlier observation that the INO80 complex lacking the Arp8 has only partial DNA binding activity, and that a recombinant complex consisting of the HSA domain of Ino80, actin, Arp4, and Arp8 binds to DNA [7], [11].

In our analysis, the Arp8 mutant Arp8 Δ1–38, which lacked the N-terminal extension, was unable to cause any shift in the DNA mobility (Fig. 1C, lanes 7 to 12), indicating that this extension is required for the stable binding of Arp8 to DNA. We have also tested the DNA binging activity of the Arp8 Δ403–463 deletant (lacking insert IV), and found that this insertion is not required for the DNA binding (Fig. 1C, lanes 13 to 18). However, the positions of DNA bands shifted by the Arp8 Δ403–463 deletant (lacking insertion IV) differed from those shifted by the wild-type Arp8, and a portion of the DNA remained at the origin. Although it remains to seen whether this observed differences in shifts is due to an alteration in the properties of the bound protein, it is likely that the altered shift reflects some involvement of the insertion IV in forming a proper Arp8-DNA complex. These observations suggest that multiple regions, protruding from the actin fold of Arp8, might contribute to the formation and/or properties of the Arp8-DNA complex in different manners.

### Arp8 binds to single-stranded DNA preferentially

**Figure 2:**
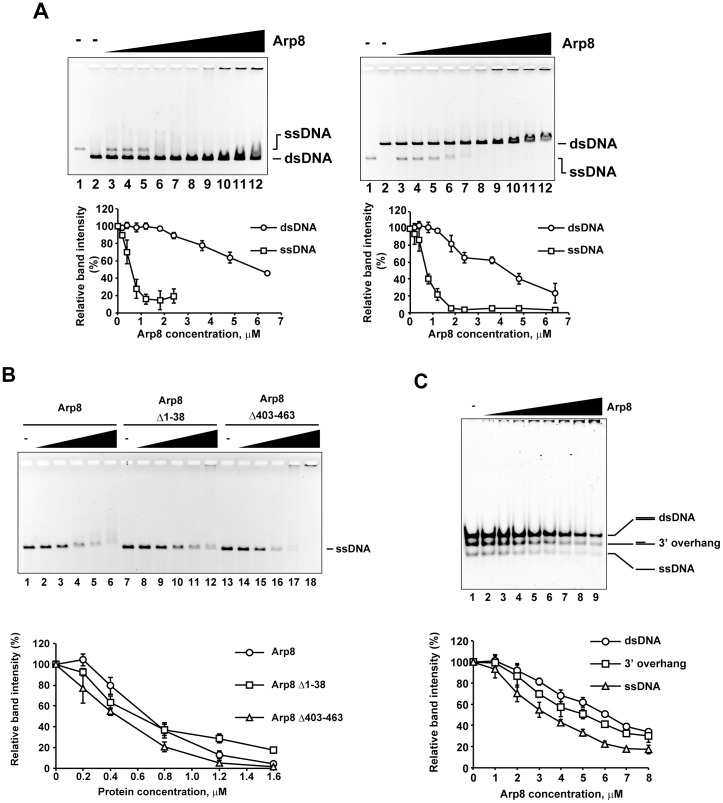
Binding of single-stranded DNA to Arp8. (A) Comparison of the binding of ssDNA (20 µM) and dsDNA (20 µM) to Arp8. Lanes 1 and 2 contain only ssDNA and dsDNA, respectively, but no protein. Lanes 3, 4, 5, 6, 7, 8, 9, 10, 11, and 12 contain 0, 0.2, 0.4, 0.8, 1.2, 1.8, 2.4, 3.6, 4.8, and 6.4 µM of Arp8, respectively. Positions of protein-free dsDNA and ssDNA were shown. (B) Binding of ssDNA to Arp8 and its deletants. Bindings of Arp8 (lanes 2–6), Arp8 Δ1–38 (lanes 8–12), and Arp8 Δ403–463 (lanes 14–18) to linearized φX174 were examined at various protein concentrations: 0 µM (lanes 1, 7, and 13), 0.2 µM (lanes 2, 8, and 14), 0.4 µM (lanes 3, 9, and 15), 0.8 µM (lanes 4, 10, and 16), 1.6 µM (lanes 5, 11, and 17), and 3.2 µM (lanes 6, 12, and 18). (C) Competitive binding of Arp8 to dsDNA, 3′-overhang DNA, and ssDNA (3 µM each). Lanes 1–9 contain 0, 1, 2, 3, 4, 5, 6, 7, 8 µM of Arp8 protein, respectively. Positions of protein-free dsDNA, 3′-overhang, ssDNA are shown. Quantification of each gel: intensity of the unbound DNA in each lane was quantified and then plotted as relative intensity (%) with respect to that of the unbound DNA from the control (no protein added control) lane.

During the HR repair process, the INO80 complex is involved in effective DNA end resection [14], [20], [21]. Therefore, we thought that Arp8 could also bind to single-stranded DNA (ssDNA), which is produced in the proximal regions of DSB by DNA end resection. Gel shift assay revealed that indeed Arp8 forms a stable complex with the ssDNA (Fig. 2, A and B). Remarkably, in the presence of same molar ratios of shorter and longer dsDNAs, the unbound fraction of ssDNA disappeared earlier than the unbound dsDNAs (Fig. 2A). These results suggest that Arp8 binds preferentially to ssDNA and that this property likely contributes to recruiting the INO80 complex to the DSB sites (see below).

As was found with the dsDNA, the Arp8 Δ403–463 deletant bound to ssDNA similarly as the full-length Arp8 (Fig. 2B, lanes 13 to 18). Although the Arp8 Δ1–38 mutant, which lacked the N-terminal extension, caused a shift in the DNA mobility (Fig. 2B, lanes 7 to 12), the apparent shift was less than that was observed for the dsDNA (Fig. 1C, lanes 7 to 12). These results suggest that the contribution of these protruding regions to the binding of ssDNA is probably similar, but not same, as their contribution to the binding of dsDNA.

During the HR process, DNA resection of the DSB generates ssDNA with 3′-overhangs and this gap between the ssDNA and dsDNA becomes the target of further DNA resection. Since the INO80 complex was reported to be involved in DNA resection, we tested a possibility that Arp8 has high affinity for DNA fragments with 3′-overhangs. The binding of Arp8 to 3′-overhang DNA was analyzed by a competitive gel shift assay in which the molar ratios of dsDNA and ssDNA were same (Fig. 2C). Results shown in Fig. 2C (plot below the gel panel) suggested that Arp8, which has a binding preference for ssDNA, binds to the 3′-overhang DNA with an apparent affinity that is in between its affinity for ssDNA and dsDNA). This result suggests that Arp8 binds to the gap between the ssDNA and dsDNA with an affinity that was less or similar to that of the ssDNA.

### ATP affects the DNA binding activity of Arp8

**Figure 3:**
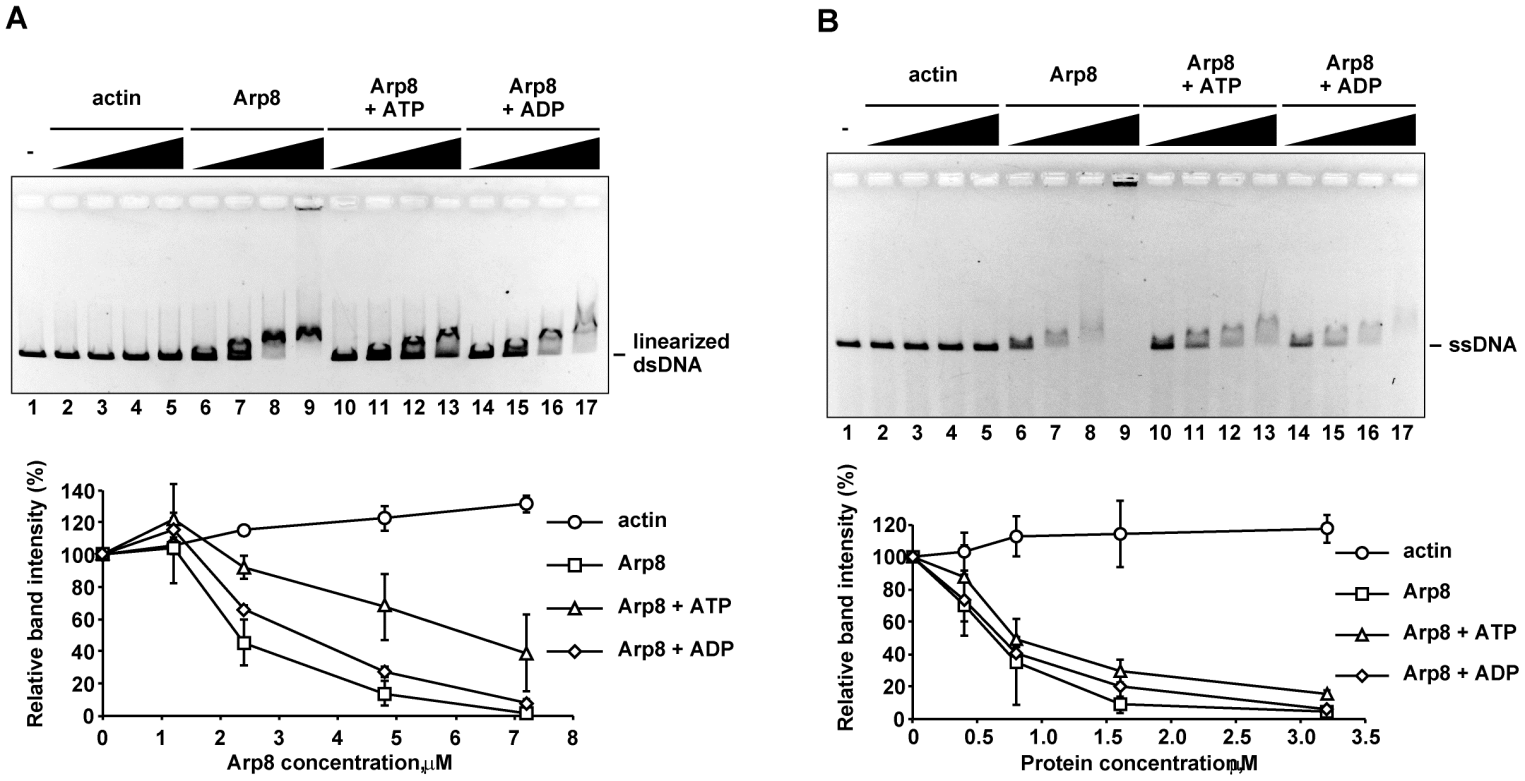
Effect of ATP on the binding of Arp8 to DNAs. Bindings of actin (lanes 2 to 5) and Arp8 (lanes 6 to 13) to dsDNA (A) and ssDNA (B) were examined in the absence (lanes 2 to 5 and 10 to 13) and presence (lanes 6 to 9) of 1 mM ATP, or in the presence of 1 mM ADP (lanes 14 to 17). Various concentrations of Arp8 (lanes 6–17) and actin (lanes 2–5) were used in this experiment. Concentrations of Arp8 used in panel (A) were: 1.2 µM (lanes 2, 6, 10, and 14), 2.4 µM (lanes 3, 7, 11, and 15), 4.8 µM (lanes 4, 8, 12, and 16), and 7.2 µM (lanes 5, 9, 13, and 17). Concentrations of Arp8 used in panel (B) were: 0.4 µM (lanes 2, 6, 10, and 14), 0.8 µM (lanes 3, 7, 11, and 15), 1.6 µM (lanes 4, 8, 12, and 16), and 3.2 µM (lanes 5, 9, 13, and 17). Lane 1 in both A and B: no protein added control. Intensity of the unbound DNA in each panel was quantified and plotted as before (see Fig. 2 legend).

It was reported earlier that ATP affects the dsDNA binding activity of the budding yeast INO80 complex *in vitro* [7]. Arp8, which shares the conserved ATP-binding pocket of the actin fold with actin, has been reported to have ATP binding activity [9], [10]. We therefore examined the effect of ATP and ADP on the DNA binding activity of Arp8. Addition of ATP to the binding assay mixture significantly decreased the binding of Arp8 to linearized dsDNA (Fig. 3A, lanes 10 to 13) and circular dsDNA (Fig. S2, lanes 7 to 12). In contrast, addition of ADP only slightly decreased the binding of Arp8 to dsDNAs (Fig. 3A, lanes 14 to 17; and Fig. S2, lanes 13 to 18). The rabbit actin exhibited significantly low DNA binding activity under the same experimental condition (Fig. 3A, lanes 1 to 5). On the other hand, addition of ATP only slightly decreased the binding of Arp8 to ssDNA (Fig. 3B, lanes 10 to 13), whereas addition of ADP seemed to have little or no effect on the binding of Arp8 to ssDNA (Fig. 3B, lanes 14 to 17). These results suggested the possibility that ATP, but not ADP, may be a regulator of the DNA binding activity of Arp8.

**Figure 4:**
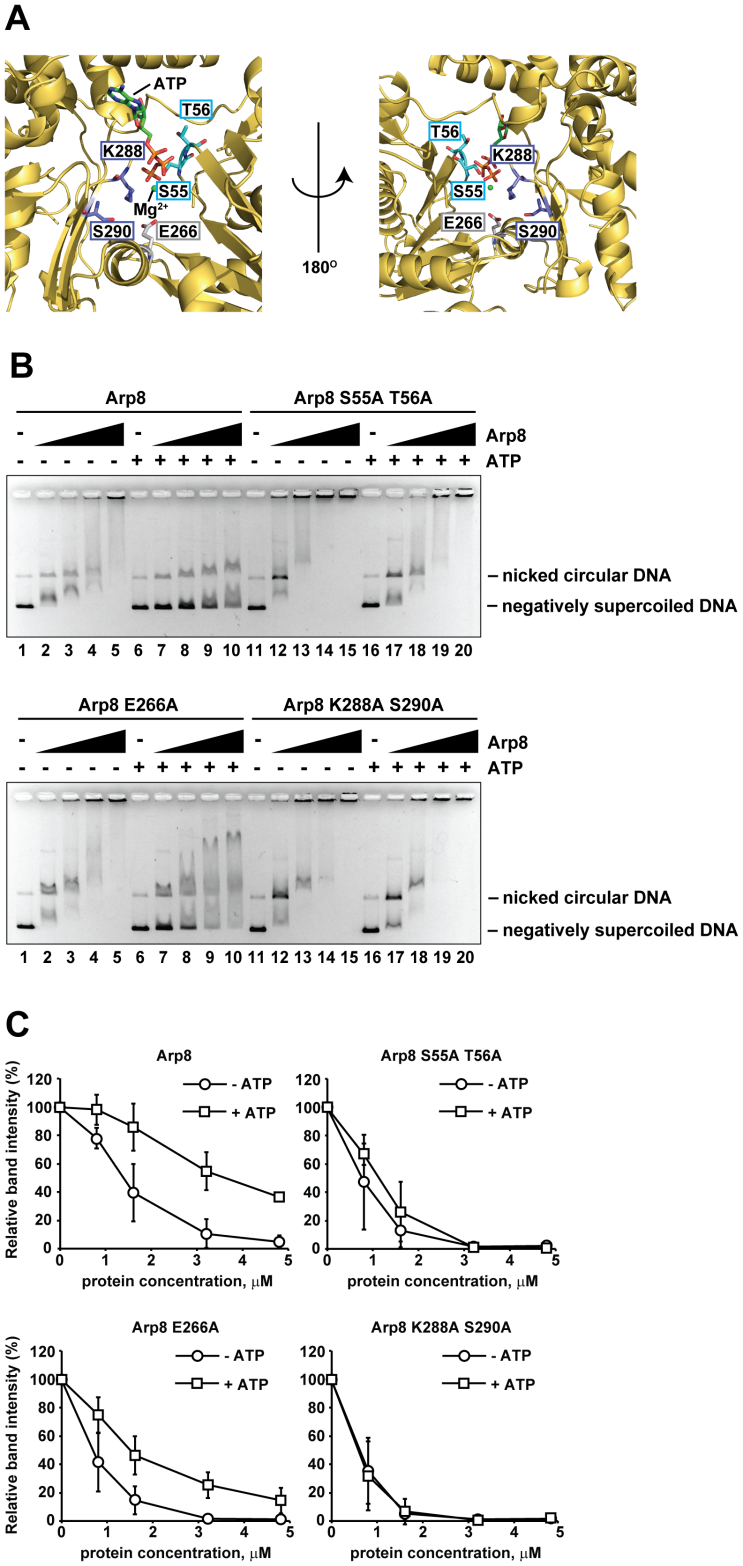
Binding of Arp8 mutants to DNAs. (A) Positions of mutations introduced in the ATP binding pocket of Arp8. The crystal structure of the ATP binding pocket of Arp8 was obtained from the Protein Database (PDB ID: 4FO0). (B) Binding of Arp8 and Arp8 mutants to supercoiled and nicked circular forms of φX174. Following concentrations of Arp8 (upper panel, lanes 1 to 10), Arp8 S55A T56A (upper panel, lanes 11 to 20), Arp8 E266A (lower panel, lanes 1 to 10), and Arp8 K288A S290A (lower panel, lanes 11 to 20) were used for this experiment: 0 µM (lanes 1, 6, 11 and 16), 0.8 µM (lanes 2, 7, 12 and 17), 1.6 µM (lanes 3, 8, 13 and 18), 3.2 µM (lanes 4, 9, 14 and 19), 4.8 µM (lanes 5, 10, 15 and 20). In lanes 6 to 10 and 16 to 20, 1 mM ATP was added to the reaction mixture. (C) Intensity of the unbound DNA in the absence or presence of ATP for each Arp8 protein (wild-type Arp8 and Arp8 mutants Arp8 S55A T56A, Arp8 E266A, and Arp8 K288A S290A) used in panel B was quantified and then the data was plotted as relative intensity (%) with respect to that of the unbound DNA from the control (no protein added control) lane.

To study the contribution of the ATP binding pocket of Arp8 in DNA binding, we designed and prepared three Arp8 mutants, namely Arp8 S55A T56A, Arp8 E266A, and Arp8 K288A S290A, by replacing the S55/T56, E266, or K288/S290 residue(s) with an Ala, respectively (Fig. 4A and Fig. S3). We chose these amino acids for the mutation analysis because they are expected to be positioned in or around the ATP-binding pocket of Arp8. In the absence of ATP, all three Arp8 mutants bound to the dsDNA (Fig. 4B, lanes 11 to 15 of upper panel, and lanes 1 to 5 and lanes 11 to 15 of lower panel, respectively) in the same manner as the wild-type Arp8 (Fig. 4B, lanes 1 to 5 of upper panel). However, in the presence of ATP, the DNA-binding activities of these Arp8 mutants were clearly different from that of the wild-type Arp8. Thus, addition of ATP significantly inhibited the DNA binding activity of the wild-type Arp8 (Fig. 4B, lanes 6 to 10), and consequently the amount of unbound DNA was increased in the presence of ATP (Fig. 4C, Arp8). In contrast, addition of ATP did not inhibit the DNA binding activities of the Arp8 S55A T56A and Arp8 K288A S290A mutants (Fig. 4B, lanes 16 to 20 of upper and lower panels, respectively), and as a result the amounts of free DNA hardly or slightly increased for these two mutants (Fig. 4B, Arp8 S55A T56A and Arp8 K288A S290A). However, addition of ATP only partially inhibited the DNA binding activity of the Arp8 E266A mutant (Fig. 4B, lanes 6 to 10 of lower panel, and Fig. 4C, Arp8 E266A). Taken together, these results suggest that the S55, T56, K288, and S290 residues of Arp8, all of which are located in the ATP binding pocket, might play some regulatory role in the binding of Arp8 to DNA in the presence of ATP.

### Establishment of Arp8-overexpressing and Arp8-knockout human cell lines

**Figure 5:**
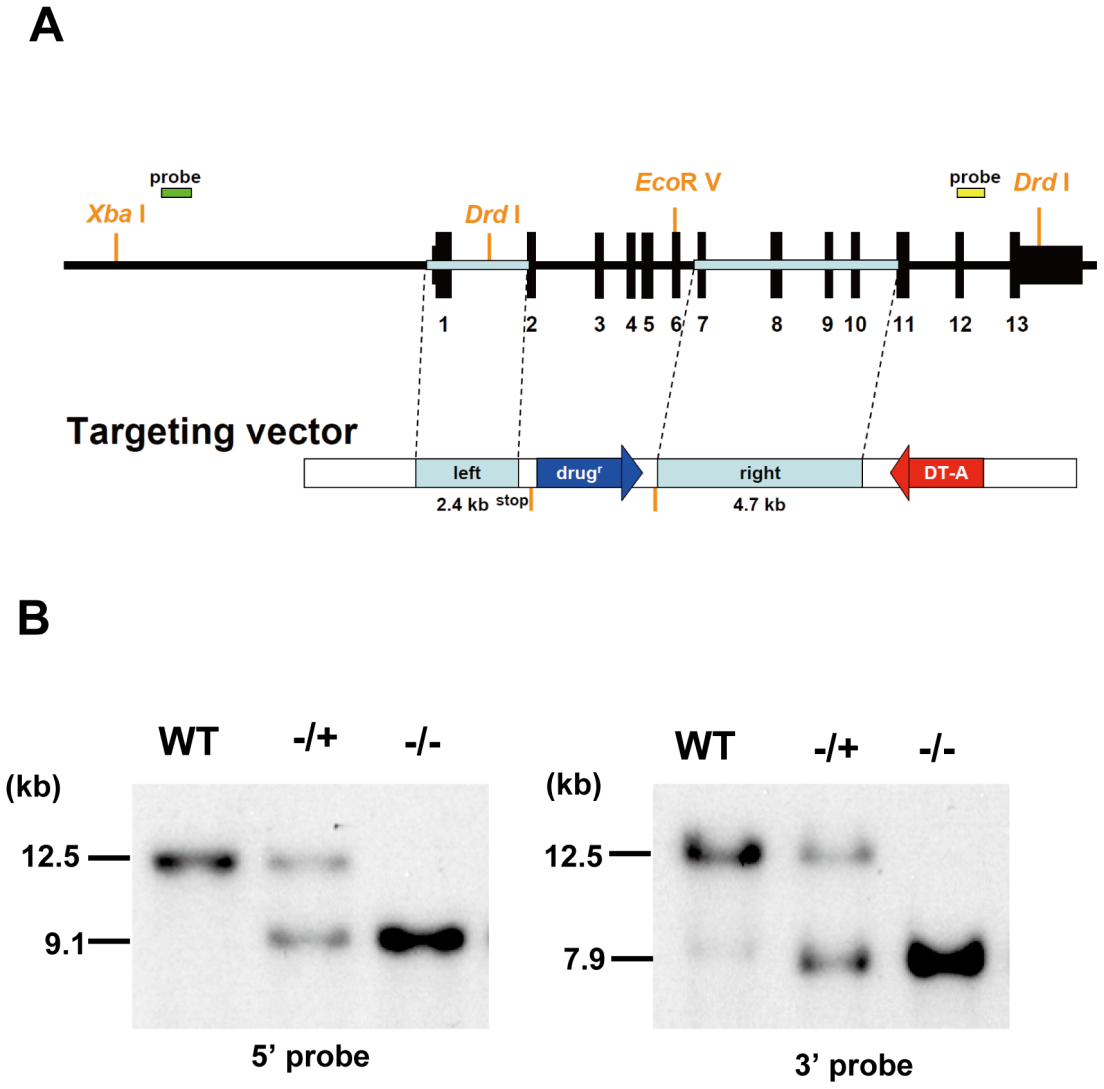
Generation of *ARP8* gene knockout in Nalm-6 cells. (A) Restriction map of the knockout construct used for the targeted disruption of the *ARP8* gene in Nalm-6 cells. Filled boxes (black) over the construct indicate positions of exons. This targeted construct was expected to disrupt five exons of *ARP8* and stop translation before the second exon. Positions of the 5′ and 3′ probes used for the Southern blot analysis and positions of the restriction enzyme sites are also indicated. (B) Restriction enzyme analysis of the targeted integration of the *ARP8* knockout construct in Nalm-6 cells. Genomic DNAs, prepared from the WT Nalm-6 cells and cells obtained after the first (+/−) and second (−/−) rounds of targeting, were digested with *Xba*I/*Eco*RV (left) or *Drd*I (right). Disruption of the *ARP8* gene was confirmed by Southern blot hybridization using the 5′ (left panel) and 3′ DNA probes (right panel) indicated in A.

**Figure 6:**
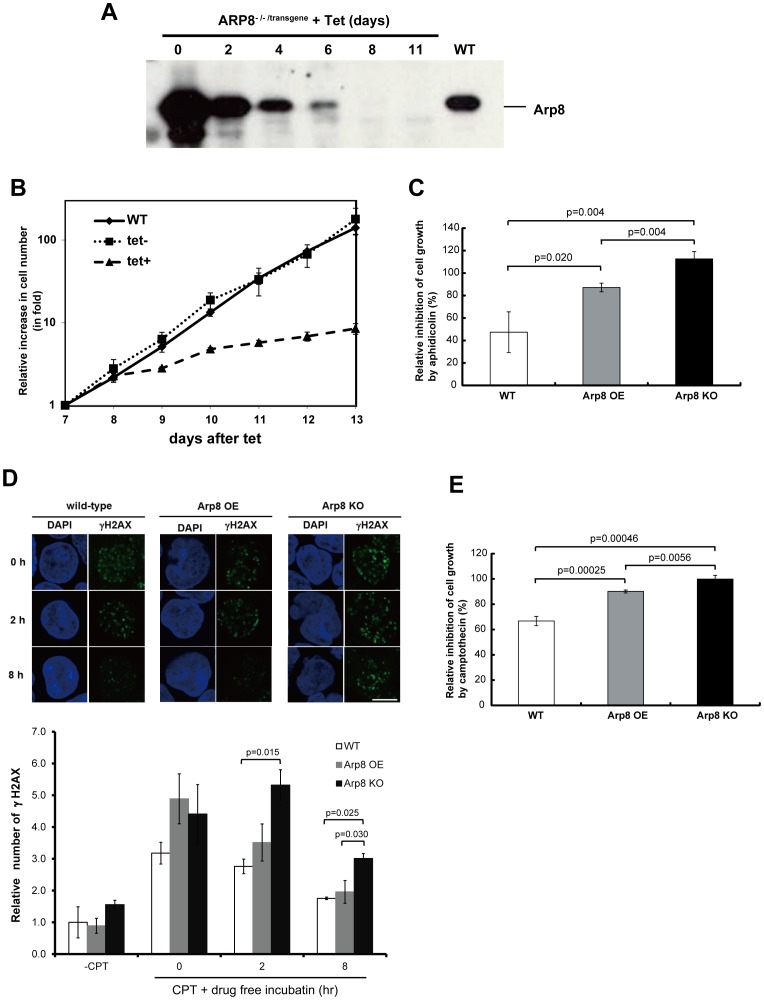
Characterization of Arp8-knockout cells. (A) Whole-cell extracts were prepared from same number of wild-type (WT) and *ARP8* ^-/-/transgene^ cells at the indicated times after the addition of 2 µg/ml of tet, and subsequently they were analyzed by Western blot using an anti-Arp8 antibody. (B) Representative growth curves for the WT and *ARP8* ^-/-/transgene^ cells with (tet+) or without (tet−) tetracycline treatment. Results shown are using cells from day 7 to day 13 after the addition of tetracycline. The number of living cells was counted after trypan blue staining and represented as fold increase in cell number. (C) Sensitivity of WT, Arp8 OE and Arp8-knockout (Arp8 KO; *ARP8* ^-/-/transgene^ cells cultured in the presence of tet for 8 days) cells to aphidicolin. Cells were cultured in the absence or presence of 0.25 µM aphidicolin for 48 h. The relative inhibition of increase in cell number by aphidicolin (%) was calculated as follows: [{(cell number at 48 h – cell number at 0 h) in the absence of aphidicolin – (cell number at 48 h – cell number at 0 h) in the presence of aphidicolin} ×100]/[(cell number at 48 h – cell number at 0 h) in the absence of aphidicolin]. If the cells did not grow at all in the presence of aphidicolin, then the (cell number at 48 h – cell number at 0 h) in the presence of aphidicolin becomes zero and the relative inhibition becomes 100%. However, sometimes in the presence of aphidicolin there were less number of cells at 48 h than at 0 h (instead of an increase in cell number), in which case the (cell number at 48 h – cell number at 0 h) becomes negative and the relative inhibition becomes more than 100%. (D) Comparison of γ-H2AX foci in wild-type, Arp8 OE, and Arp8 KO cells. The cells were treated with camptothecin (CPT) for 1 h, and after washing out the reagent, the cells were incubated without CPT for 2 h or 8 h. Immunostained γ-H2AX foci were observed under a fluorescence microsope, and the number of foci was counted using the ImageJ software. Plot below shows the number of γ-H2AX foci in the indicated cells relative to that in the camptothecin-untreated (-CPT) wild-type cells. (E) Wild-type, Arp8 OE, and Arp8 KO cells were treated with 1 µM camptothecin for 1 h. After washing out the reagent, the relative inhibition by camptothecin was shown as in C. Error bars indicate average mean ± SD (n =  at least 3 independent experiments).

So far, the function of Arp8 in mammalian cells has been analyzed by knocking down its expression by RNA interference. We have developed a conditional Arp8 knockout (KO) cell line from the parent human Nalm-6 B cell line (Fig. 5) (see also Materials and Methods). In the *ARP8* ^-/-/transgene^ cells, the expression of Arp8 is under the control of a tetracycline (tet)-repressible promoter. Western blot analysis showed that Arp8 was overexpressed in *ARP8* ^-/-/transgene^ cells in the absence of tetracycline (Fig. 6A, day 0). The amount of expressed Arp8 reduced gradually after the addition of tetracycline, and expression of Arp8 was not detectable after 8-day (Fig. 6A). Since our previous study indicated that this tet-repressible promoter shuts down within a day after the tetracycline addition [22], results obtained in this study suggest that Arp8 is a relatively stable protein. Overexpression of Arp8 did not have any apparent adverse effect on the cell growth (Fig. 6B, -tet versus WT). However, following the addition of tetracycline, when Arp8 became undetectable on Western blot, a significant decline in the growth of *ARP8* ^-/-/transgene^ cells, but no immediate cell death, was observed. This result suggests that Arp8 is required for normal cell growth.

### DNA repair is impaired in Arp8-knockout cells

To test the possibility whether Arp8 may have any role in DNA repair, we treated wild-type, Arp8-overexpessed (Arp8 OE), and Arp8-knockout (Arp8 KO) cells with aphidicolin and camptothecin to induce DSBs in these cells. Although the repair process of aphidicolin- and camptothecin-induced DSBs are not closely connected to the HR-repair process of endonuclease-induced DSBs, there are some similarities between these two repair processes [23], [24], [25], [26]. Fig. 6C shows the relative inhibition of increase in number of cells grown in the presence of aphidicolin as compared to that of the untreated cells. In this plot, 0% inhibition means that the cells grew in the presence of aphidicolin to the same extent as in the absence of the drug, and 100% inhibition means that no increase in cell number was observed in the presence of aphidicolin (see legend of Fig. 6C for further explanation). In wild-type cells, aphidicolin inhibited the cell growth by 50%. In Arp8 KO cells, the value was −15%, which meant that the cell number did not increase at all in the presence of aphidicolin, but instead decreased, possibly because of apoptosis. Thus, this induced inhibition in cell growth was most severe for the Arp8 KO cells than for the wild-type and Arp8 OE cells. This result suggests that knockout of Arp8 probably impairs DNA repair, which probably takes place via the HR or an HR-like repair process.

Camptothecin-induced repair was also analyzed in wild-type, Arp8 OE, and Arp8 KO cells (Fig. 6D). For this experiment, cells were treated with camptothecin for 1 h. Cells were then washed to remove camptothecin and were incubated for up to 8 h without the drug. Induced DSBs were analyzed by comparing the relative number of γ-H2AX foci. As shown, in all three cells the observed number of γ-H2AX foci was not significantly different at 0 h of the drug free incubation (Fig. 6D, 0 h). However, the number of γ-H2AX foci in Arp8 KO cells, observed after 2 h and 8 h of drug free incubation, respectively, was significantly higher than the number of foci observed in the wild-type and Arp8 OE cells (Fig. 6D, 2 h and 8 h), suggesting that the repair of camptothecin-induced DSB is impaired in the absence of Arp8. Consistent with this observation, the camptothecin-induced inhibition in cell growth was most severe for the Arp8 KO cells than for the wild-type and Arp8 OE cells (Fig. 6E). The experiment to determine sensitivity to camptothecin was performed using Arp8 KO cells that were treated with tetracycline for eight days, and the results were normalized with respect to the Arp8 KO cells that were not exposed to camptothecin. These observations support the idea that Arp8 contributes to the progression of DNA repair which probably takes place via the HR or an HR-like repair process.

## Discussion

Our study suggested that the binding of human Arp8 to both dsDNA and ssDNA is relatively stable. This is the first example demonstrating the binding of dsDNA and ssDNA to one of the components of the INO80 complex and also to a member of the actin family of proteins. Recently, three dimensional architecture of the budding yeast INO80 complex has been determined by cryo-electron microscopy [11]. Accordingly, the INO80 complex is organized in four modules (head-neck-body-foot architecture). Among these modules, Arp8 forms the foot module together with Arp4, another histone-binding Arp, and this module is called as the Arp8 module [11]. When the INO80 complex binds to chromatin, a nucleosome is captured and placed between the Arp8 module and the head module. As the link between the Arp8 module and body module is flexible, it allows the Arp8 module to fold back and stabilize the nucleosome [9], [11]. It is thought that both Arp4 and Arp8 contribute to the folding back of the Arp8 module through their histone binding activities. In addition, it has been shown that actin in the Arp8 module is involved in associating the INO80 complex with the extranucleosomal DNA [27]. Thus, the binding of Arp8 to the DNA adjacent to a nucleosome, together with the histone binding activity of Arp8, is expected to stabilize the binding of INO80 complex to the nucleosome.

The INO80 complex lacking Arp8 showed a moderate, but not a complete, decrease in the DNA binding activity [11]. This observation suggested that some other component(s) of the complex might also have DNA binding activity. It is known that the ATPase activity of the INO80 complex is stimulated in the presence of DNA, and the DNA-stimulated ATPase activity is completely abolished in the INO80 complex lacking Arp8 [11]. Interestingly, the INO80 complex lacking Arp8 is stimulated by nucleosome. Thus, our observations are consistent with the model that the DNA binding activity of Arp8 contributes to the DNA-stimulated INO80 functions. However, further analyses are necessary to clarify this issue fully.

The INO80 complex is recruited to double-strand breaks (DSBs) [14], [15]. At these sites, γ-H2AX (phosphorylated-H2A in yeast and phosphorylated-H2AX in mammals) is accumulated as a signal for recruiting protein factors. In yeast, Arp4 and Nhp10 interact with γ-H2AX and are required for the recruitment of the INO80 complex. However, human Arp4 is not required for the recruitment of the complex to DSB, and Nhp10 is not conserved in human INO80 complex [17]. In yeast, DNA end resection is required for recruiting the INO80 complex [28]. Although the need for ssDNA in recruiting the human INO80 complex to DSB is not yet fully understood [16], [17], [28], it is worth noting that Arp8 is indispensable for recruiting the human INO80 complex to DSBs [17]. Therefore, it is likely that the ssDNA-binding activity of Arp8 contributes to the process of recruiting INO80 complex to DSBs.

Our results indicated that the DNA binding activity of Arp8 decreased in the presence of ATP. Previously, it was observed that ATP affects the intra-molecular interactions in budding yeast Arp4 [5]. It was also proposed that the binding of ATP to Arp4 promotes disassembly of Arp4 from the chromatin remodeling and modification complexes [3], [5]. In this respect, Arp4 seems to have some similarity to actin, whose monomer-filament transition is regulated by ATP-binding. Since the INO80 complex contains multiple actin-family proteins, such as Arp4, Arp8, and actin as well [11], [29], it is plausible that the binding of ATP to these actin family proteins regulates the function of the INO80 complex by affecting their intramolecular interactions. The actin fold consists of two major domains, and the relative configuration of these major two domains is shifted as a result of ATP binding [30], [31], [32]. Since the mutational analyses of the ATP binding pocket of Arp8 suggested that the binding of ATP to Arp8 play an important role in DNA binding (Fig. 4B), it is likely that the binding of ATP to Arp8 change the relative configuration of these two domains and thereby affect the DNA binding activity.

**Figure 7:**
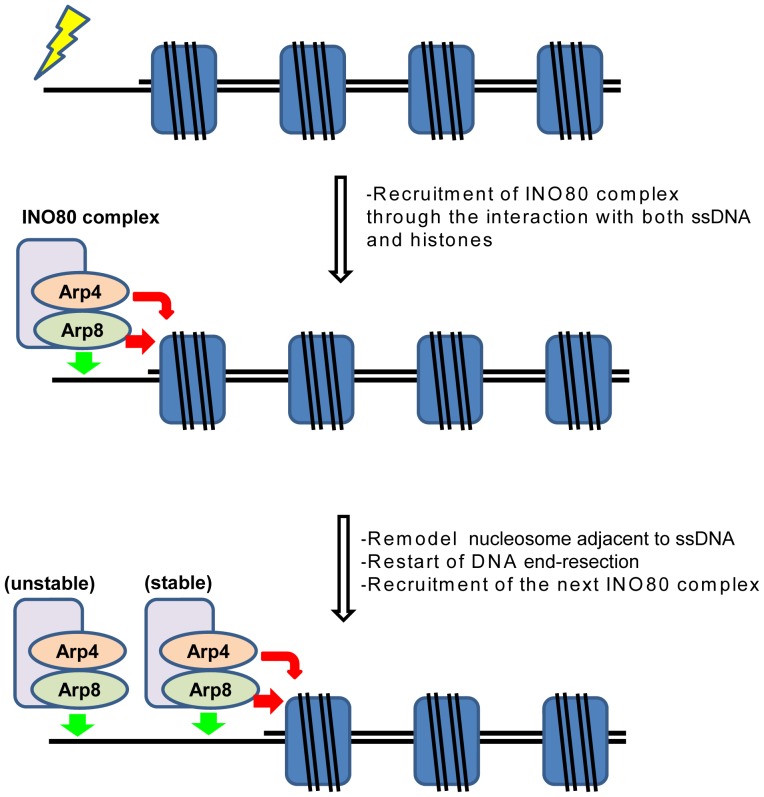
A schematic model depicting the role of Arp8 in the early stage of HR repair. Green and red arrows near the INO80 complex represent binding of Arp8 to ssDNA and binding of Arp4 and Arp8 to histones, respectively. See text for details.

In both budding yeast and human, the INO80 complex is required for the efficient DNA end section during HR repair [14], [15]. The INO80 complex is recruited to the DSB site in the early stage of the HR repair process. Importantly, disruption of the Arp8 gene in the budding yeast has caused a defect in HR repair [33]. Based on our results, we have proposed a model depicting how Arp8 might contribute to the function of the INO80 complex during the HR repair (Fig. 7). After DSB occurs, endonucleases start DNA end resection. Nucleosomes proximal to the DSB site pose obstacles for the DNA end resection by endonucleases, and this nucleosome barrier could halt the formation of ssDNA (Fig. 7, first row). The ssDNA binding activity of Arp8, together with the histone binding activities of Arp4 and Arp8, would facilitate the binding of INO80 complex to the nucleosomes flanking the ssDNA (Fig. 7, second row). This recruited INO80 complex could then evict or reposition the adjacent nucleosome, and this process may be required to overcome the nucleosome barrier in order for the DNA end resection to progress (Fig. 7, second row). After evicting or relocating the first nucleosome, the newly resected ssDNA adjacent to the next nucleosome barrier is targeted by the second INO80 complex (Fig. 7, third row). The first INO80 complex stays bound to the original position on the DNA through its own ssDNA binding activity, but without associating with histones (Fig. 7, third row). This model is consistent with earlier observations that knockdown of Arp8 impairs RPA focus formation and that knockdown of Ino80, although affects an early event, is not necessary for the later stages of HR repair [15].

Alternatively, since Arp8 also has INO80 complex-independent function at least in mitotic chromosome segregation [34], DNA-binding activity of Arp8 might contribute to DNA repair independently of the INO80 complex. Further analyses would be necessary to clarify the mechanism of Arp8 in DNA repair.

It was previously shown that the DNA damage-induced stimulation of poly (ADP-ribose) polymerase led to a decrease in the ATP pool [35]. Since the DNA-binding ability of Arp8 is relatively high under an ATP-deprived condition (Fig. 2), this change in ATP concentration could also be involved in the function of Arp8 in DNA repair.

DNA damage repair is crucial for the maintenance of genome stability and cancer suppression. Therefore, defects in DNA repair could be relevant to human diseases. Recently, an association was found between the SNPs in the *INO80* gene and chronic kidney disease (CKD), an important public health problem with a genetic component [36]. Indeed, Zhou et al. (2012) have recently shown that inadequate DNA repair is relevant to CKD. Since Arp8 is essential for the proper functioning of the INO80 complex, its dysfunction in human cells would be expected to cause diseases. By using our tet-inducible Arp8-knockout cell line, we would be able to analyze the effects of Arp8 expression level on genome stability and would also be able to analyze functions of Arp8 mutants in the absence of endogenous Arp8. This system will provide further knowledge on the roles of Arp8 and INO80 complex in epigenetic regulations including DNA repair and transcription.

## Materials and Methods

### Expression and purification of proteins

Human Arp8 was overexpressed in *Escherichia coli* cells as an N-terminal hexahistidine (His6)-tagged protein. To achieve this, the full length wild-type (WT) or mutagenized Arp8 (Arp8 Δ1–38, Arp8 Δ403–463, Arp8 S55A T56A, Arp8 E266A, and Arp8 K288A S290A) coding cDNA fragment was ligated into the pET15b vector (Novagen), which harbors a His_6_ tag and a PreScission protease-recognition sequence (GE Healthcare Bioscience) at the N-terminus. Primers used for the site-directed mutagenesis are shown in Table S1. Recombinant Arp8 was expressed in *E. coli* and purified as follows. Briefly, *E. coli* cells carrying the Arp8 expression plasmid, grown on ampicilin (100 µg/ml) and chloramphenicol (35 µg/ml) supplemented LB plates at 37°C, were used for inoculating 5 l growth medium (LB containing 100 µg/ml ampicillin) and the culture was grown with shaking at 37°C. When the cell density reached an OD600 of 0.45–0.55, 1 mM isopropyl beta-D-1-thiogalacropryranoside (IPTG) was added to induce the expression of Arp8 and the culture was further incubated at 18°C for 15 h. Cells were harvested, resuspended in 30 ml of buffer A [50 mM Tris-HCl (pH 8.0), 0.7 M NaCl, and 10% glycerol] containing 1× Protease Inhibitor Cocktail (Nakalai Tasque) and disrupted by sonication. After removing cell debris by centrifugation, the lysate was mixed with 2 ml (50% slurry) of Ni-NTA beads. After packing the beads in an Econo-column (BioRad), the bound His6-tagged Arp8 was eluted by using a linear gradient of imidazole in 50 mM NaCl (pH 8.0), 0.1 M NaCl, and 10% glycerol. Fractions containing His6-Arp8 were identified by SDS-PAGE, and combined fractions were applied to a Heparin Sepharose column (GE Healthcare Biosciences). After washing the column with buffer B [20 mM Tris-HCl (pH 8.0), 0.1 M NaCl, 0.25 mM EDTA, 2 mM 2-mercaptoethanol and 10% glycerol], Arp8 was eluted using a linear gradient of 100 to 1,000 mM NaCl. Fractions containing Arp8 were collected and treated with PreScission protease (3 units/mg of protein) to remove the His6 tag. The resultant Arp8 was further purified using a MonoQ column (GE Healthcare Bioscience), from where the bound Arp8 was eluted with a gradient of 0 to 0.5 M NaCl in buffer C [20 mM Tris-HCl (pH 8.0), 0.25 mM EDTA, 2 mM 2-mercaptoethanol and 10% glycerol], and the eluted protein was dialyzed against buffer G [20 mM Tris-HCl (pH 8.0), 0.2 M NaCl, 0.25 mM EDTA, 2 mM 2-mercaptoethanol and 10% glycerol]. The concentration of purified Arp8 was determined by the Bradford method [37] with BSA as the standard protein.

### DNA binding assay

Single-stranded φX174 viral (+) strand DNA was purchased from New England Biolabs. The linear dsDNAs were prepared by digesting the φX174 replicative form I DNA and pUC19 vector with the restriction enzyme *Pst*I. The DNAs were mixed with the purified Arp8 protein in 10 µl of reaction mixture containing 20 mM HEPES-NaOH (pH 7.5), 1 mM DTT, 0.1 mg/ml bovine serum albumin, 1 mM MgCl_2_, 160 mM NaCl, 8% glycerol, and with or without 1 mM ATP. The reaction mixture was incubated at 37°C for 15 min, and was then analyzed by 0.8% agarose gel electrophoresis in 1xTAE buffer (electrophoresis was carried out at 3.0 V/cm for 2 h). The DNA bands were visualized by ethidium bromide staining.

To perform the competitive DNA-binding assay, we prepared double-stranded DNA (dsDNA), 3′-overhang DNA, and single-stranded DNA (ssDNA) as described previously (MacKay C et al., 2010). We used oligonucleotides a3, a3-cp, and c described earlier [38], whose nucleotide sequences were as follows: a3, 5′- CCTCG ATCCT ACCAA CCAGA TGACG CGCTG CTACG TGCTA CCGGA AGTCG; a3-cp, 5′- CGACT TCCGG TAGCA CGTAG CAGCG CGTCA ACTGG TTGGT AGGAT CGAGG; and c, 5′- GCCTA GAGTG CAGTT CGTGG CGAGC. To prepare dsDNA and 3′-overhang DNA, the oligonucleotide pairs a3 and a3-cp, and a3 and c, respectively, were annealed, and the annealed DNAs were then purified by polyacrylamide gel electrophoresis. The oligonucleotide a3 was used as the ssDNA. A mixture containing 3 µM each of the dsDNA, 3′-overhang DNA, and ssDNA was incubated with the indicated amounts of Arp8 at 37°C for 15 min in 10 µl of 20 mM HEPES-NaOH buffer (pH 7.5) supplemented with 250 mM NaCl, 1 mM MgCl_2_, 0.1 mg/ml bovine serum albumin, and 1 mM DTT. The protein-DNA complexes were separated by electrophoresis on 10% polyacrylamide gel in 0.5xTBE buffer (at 6.25 V/cm for 150 min), and were visualized after staining with SYBR Gold (Invitrogen).

### Cell culture and cell viability analyses

Nalm-6 cells were cultured at 37°C in Roswell Park Memorial Institute medium containing GlutaMAX-I (Invitrogen) supplemented with 10% fetal calf serum, penicillin, and streptomycin as described earlier (Ono et al., 2009). To suppress the expression of the tetracycline (tet)-responsive Arp8 transgene, tetracycline (Sigma) was added to the culture medium to a final concentration of 2 µg/ml. The number of viable cells after the addition of aphidicolin (Wako) or camptothecin (Wako) was counted by the trypan blue dye exclusion assay.

### Establishment of Arp8-deficient cells by using a tetracycline (Tet)-regulated gene depletion (Tet-Off) system

Tet-Off Arp8 cells were established following a protocol described previously [22], [39]. In brief, the full length cDNA of *ARP8* was cloned into the pTRE-IRES-neo vector to yield a tetracycline-regulated Arp8 expression plasmid. The left (2.4 kb) and right (4.2 kb) arms of the targeting vector were respectively amplified by genomic PCR. The left arm and the right arm contained exon 1 and exons 7–10, respectively (see Fig. 5A). These fragments were used to generate the final targeting plasmids, pTARGET-*ARP8*-His and pTARGET-*ARP8*-Puro. Gene targeting and screening of purposive clones were performed as described previously [22], [39]. The disruption of both alleles of *ARP8* gene was confirmed by Southern blot analysis using probes shown in Fig. 5A and the results are shown in Fig. 5B. Established Arp8 Tet-Off cells were confirmed by Western blot analysis using an anti-Arp8 antibody.

### Indirect immunofluorescence staining and Western blot analysis

Cells were fixed in 4% paraformaldehyde in phosphate-buffer saline. To visualize γ-H2AX foci, an anti-γ-H2AX antibody (Milipore) was used for immunostaining, and the bound antibody was detected using a fluorescent-conjugated secondary anti-mouse antibody. Cellular DNA was stained with 1 mg/ml of DAPI, and fluorescence of bound DAPI and γ-H2AX was observed under a confocal laser scanning microscope (FV1000, Olympus). The number of γ-H2AX foci was counted by using the Image J software. Western blot analysis was performed using an anti-Arp8 antibody [34] or an anti-H3 antibody (Abcam ab1791). An anti-IgG conjugated to horseradish peroxidase (Promega) was used as the secondary antibody, and ECL Western blotting detection reagents (GE Healthcare) were used for the detection of bound antibodies [40].

## Supporting Information

Figure S1 **Schematic diagrams of full-length and deletion mutants of Arp8.** The N-terminal extension and insertions are shown in red and light blue, respectively.
(TIF)

Figure S2 **Binding of Arp8 to supercoiled and nicked circular forms of φX174 in the presence of ATP or ADP.** Binding of Arp8 was examined in the absence (lanes 1 to 6) or presence of 1 mM ATP (lanes 7 to 12), and in the presence of 1 mM ADP (lanes 13 to 18) as well. Concentrations of Arp8 used were: 0 µM (lanes 1, 7, and 13), 0.4 µM (lanes 2, 8, and 14), 0.8 µM (lanes 3, 9, and 15), 1.6 µM (lanes 4, 10, and 16), 3.2 µM (lanes 5, 11, and 17), and 4.8 µM (lanes 6, 12, and 18). Intensity of the unbound DNA band in each lane was quantified and plotted as relative intensity (%) with respect to the intensity of the unbound DNA from the control (no protein added) lane.
(TIF)

Figure S3 **SDS-PAGE analysis of purified wild-type and ATP binding pocket mutants of Arp8.** Lane 1: molecular weight markers. Lane 2: wild-type Arp8, Lane 3: Arp8 S55A T56A, Lane 4: Arp8 E266A, and Lane 5: Arp8 K288A S290A.
(TIF)

Table S1 **Oligonucleosides for cytodirected mutagenesis.**
(DOCX)
