# Supplementary figures and images for: DNA Binding Properties of the Actin-Related Protein Arp8 and Its Role in DNA Repair
Source: PLoS One. 2014 Oct 9;9(10):e108354. doi: 10.1371/journal.pone.0108354 (PMC4191963; doi:10.1371/journal.pone.0108354)

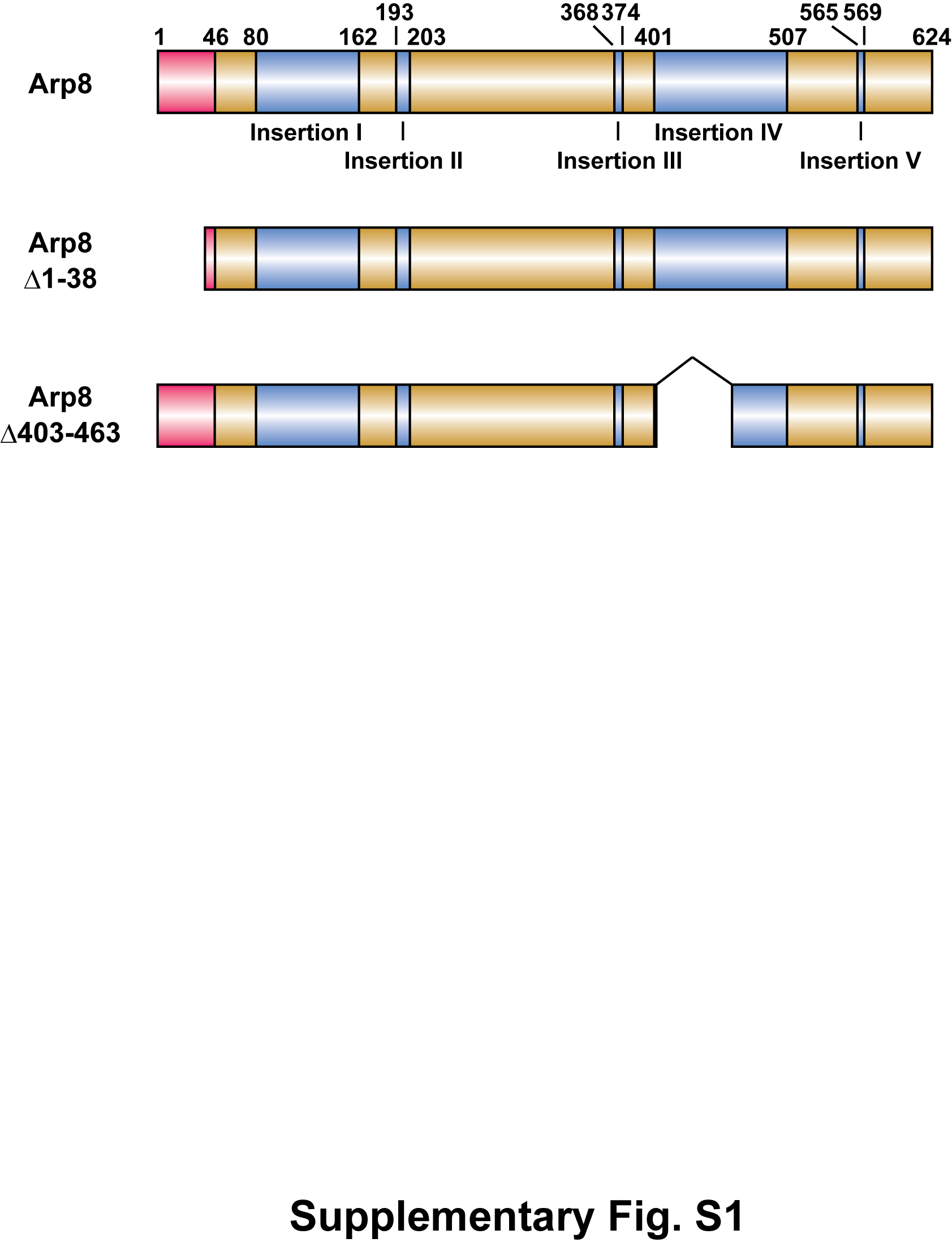

Supplement: Figure S1 — Schematic diagrams of full-length and deletion mutants of Arp8. The N-terminal extension and insertions are shown in red and light blue, respectively. (TIF) [file pone.0108354.s001.tif]

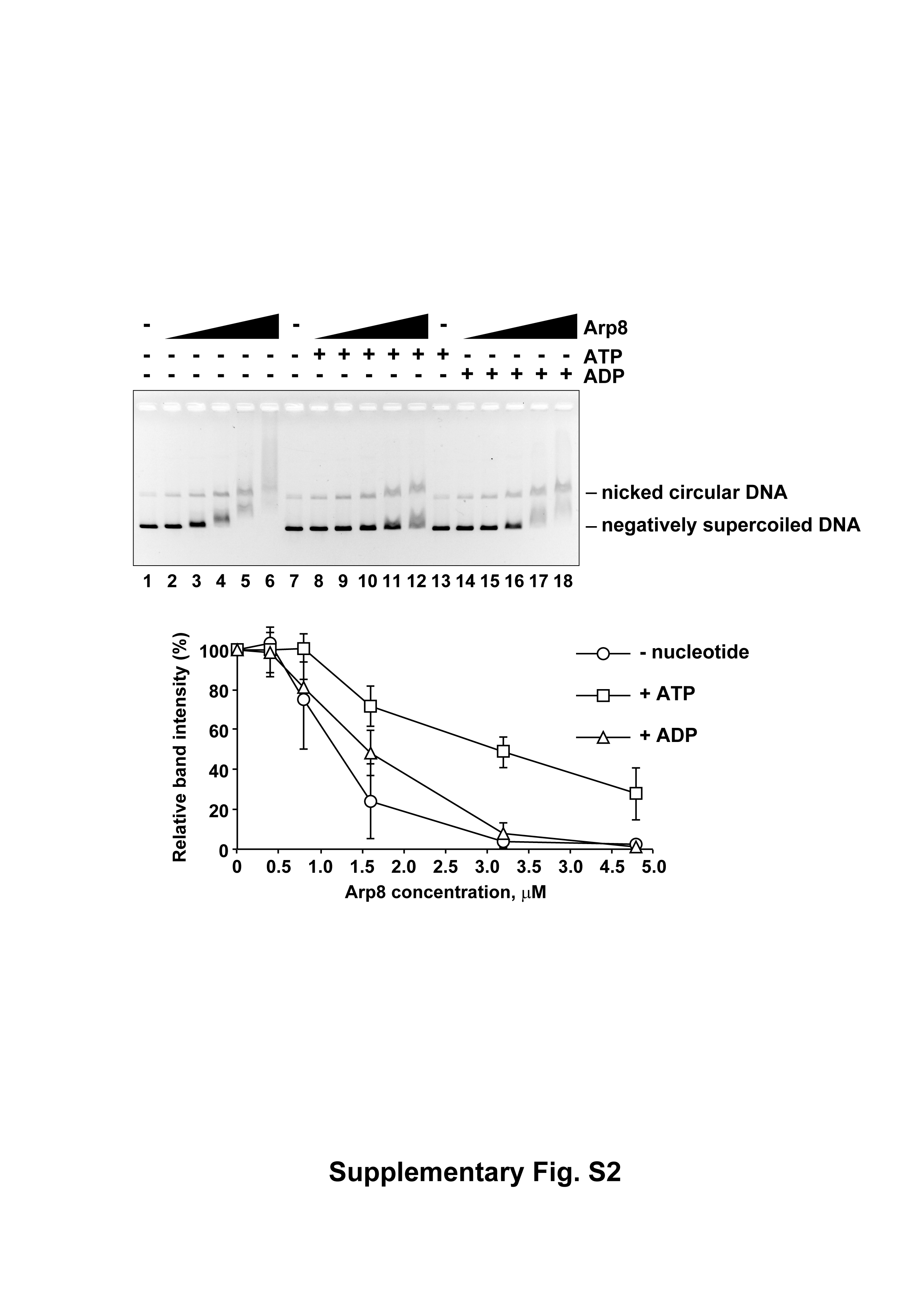

Supplement: Figure S2 — Binding of Arp8 to supercoiled and nicked circular forms of φX174 in the presence of ATP or ADP. Binding of Arp8 was examined in the absence (lanes 1 to 6) or presence of 1 mM ATP (lanes 7 to 12), and in the presence of 1 mM ADP (lanes 13 to 18) as well. Concentrations of Arp8 used were: 0 µM (lanes 1, 7, and 13), 0.4 µM (lanes 2, 8, and 14), 0.8 µM (lanes 3, 9, and 15), 1.6 µM (lanes 4, 10, and 16), 3.2 µM (lanes 5, 11, and 17), and 4.8 µM (lanes 6, 12, and 18). Intensity of the unbound DNA band in each lane was quantified and plotted as relative intensity (%) with respect to the intensity of the unbound DNA from the control (no protein added) lane. (TIF) [file pone.0108354.s002.tif]

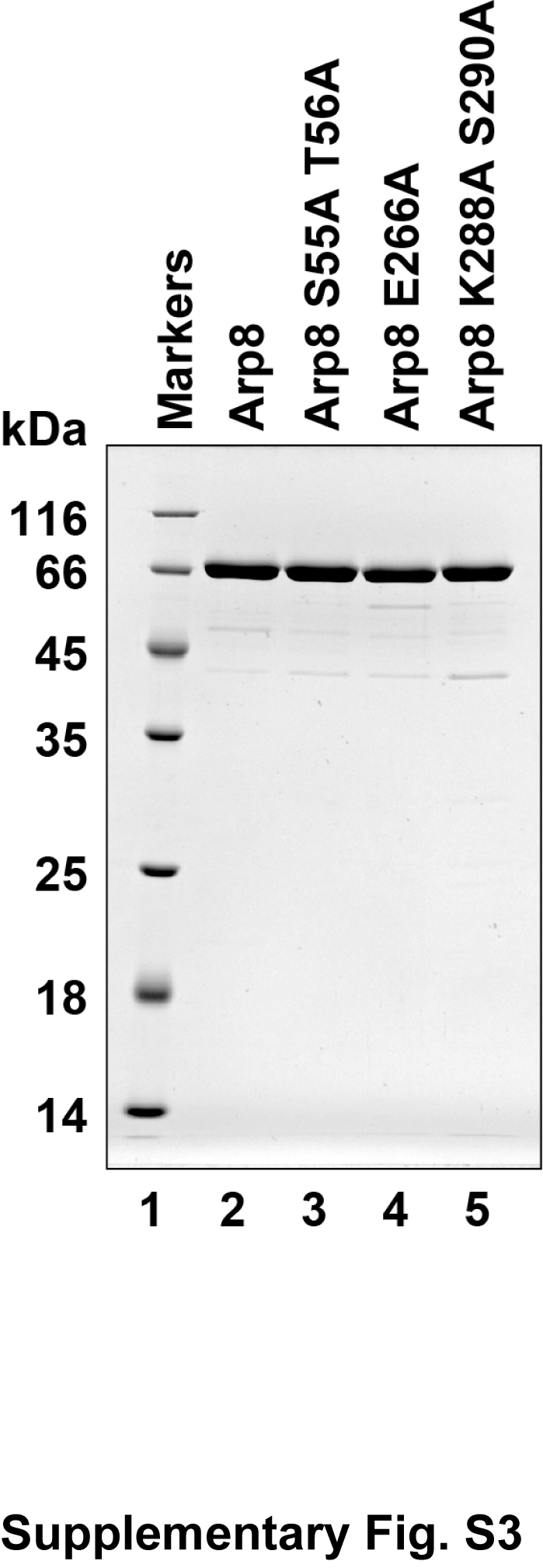

Supplement: Figure S3 — SDS-PAGE analysis of purified wild-type and ATP binding pocket mutants of Arp8. Lane 1: molecular weight markers. Lane 2: wild-type Arp8, Lane 3: Arp8 S55A T56A, Lane 4: Arp8 E266A, and Lane 5: Arp8 K288A S290A. (TIF) [file pone.0108354.s003.tif]
