# Supplementary material for: DNA Binding Properties of the Actin-Related Protein Arp8 and Its Role in DNA Repair
Source: PLoS One. 2014 Oct 9;9(10):e108354. doi: 10.1371/journal.pone.0108354 (PMC4191963; doi:10.1371/journal.pone.0108354)
Supplement: Table S1 — Oligonucleosides for cytodirected mutagenesis. (DOCX) [file pone.0108354.s004.docx]

Supplementary Table S1

Oligonucleosides for cytodirected mutagenesis

Arp8 S55A T56A top

5’ gtcatacatccaggtgcggcgactttaaggattggt 3’

Arp8 S55A T56A comp

5’ ACCAATCCTTAAAGTCGCCGCACCTGGATGTATGAC 3’

Arp8 E266A top

5’ gtggtccatcaggcgtctgtgtgtgcc 3’

Arp8 E266A comp

5’ GGCACACACAGACGCCTGATGGACCAC 3’

Arp8 K288A S290A top

5’ acagcggtatgctgtgtggaggatggggtgtct 3’

Arp8 K288A S290A comp

5’ CGCCTGGTCCCCAACGTCTACAATACACGTGCT 3’
